# Early termination does not negatively impact the outcome of adjuvant immunotherapy in melanoma

**DOI:** 10.1111/jdv.20650

**Published:** 2025-03-22

**Authors:** Dirk Tomsitz, Elisabeth Livingstone, Carmen Loquai, Martin Kaatz, Ulrike Leiter, Bastian Schilling, Patrick Terheyden, Jessica Hassel, Michael Sachse, Jens Ulrich, Edgar Dippel, Frank Meiss, Claudia Pföhler, Alexander Kreuter, Rudolf Herbst, Michael Weichenthal, Lisa Zimmer, Friedegund Meier, Ricarda Rauschenberg, Peter Mohr, Fiona Brunnert, Imke von Wasielewski, Ralf Gutzmer, Dirk Schadendorf, Carola Berking, Selma Ugurel, Lucie Heinzerling

**Affiliations:** ^1^ Department of Dermatology and Allergy University Hospital, LMU Munich Munich Germany; ^2^ Department of Dermatology University Hospital Essen Essen Germany; ^3^ German Cancer Consortium (DKTK), Partner Site Essen/Düsseldorf Essen Germany; ^4^ Department of Dermatology Gesundheit‐Nord Hospital Bremen Germany; ^5^ Department of Dermatology DRK Hospital Chemnitz‐Rabenstein Rabenstein Germany; ^6^ Division of Dermatooncology, Department of Dermatology University Medical Center Tuebingen Germany; ^7^ Department of Dermatology University Hospital Würzburg Würzburg Germany; ^8^ Department of Dermatology, Allergology and Venerology University Medical Center Schleswig Holstein Lübeck Campus Lubeck Germany; ^9^ Department of Dermatology, National Center for Tumor Diseases (NCT) University Hospital Heidelberg Heidelberg Germany; ^10^ Department of Dermatology Hospital Bremerhaven Reinkenheide Bremerhaven Germany; ^11^ Department of Dermatology and Allergy Harzklinikum Dorothea Christiane Erxleben GmbH Quedlinburg Germany; ^12^ Department of Dermatology Ludwigshafen City Hospital Ludwigshafen Germany; ^13^ Department of Dermatology and Venerology, Faculty of Medicine Medical Center – University of Freiburg Freiburg Germany; ^14^ Department of Dermatology Saarland University Hospital and Saarland University Faculty of Medicine Homburg Germany; ^15^ Department of Dermatology, Venerology and Allergology, Helios St. Elisabeth Klinik Oberhausen University Witten‐Herdecke Oberhausen Germany; ^16^ Department of Dermatology HELIOS Hospital Erfurt Erfurt Germany; ^17^ Department of Dermatology, Skin Cancer Center University Hospital Schleswig‐Holstein – Campus Kiel Kiel Germany; ^18^ Department of Dermatology University Hospital Carl Gustav Carus Dresden Germany; ^19^ Skin Cancer Center National Center for Tumor Diseases Dresden Germany; ^20^ Department of Dermatology Elbe Clinic Buxtehude Buxtehude Germany; ^21^ Skin Cancer Center Hannover, Department of Dermatology and Allergy Hannover Medical School Hannover Germany; ^22^ Department of Dermatology, Johannes Wesling Medical Center Minden Ruhr University Bochum Minden Germany; ^23^ National Center for Tumor Diseases West, Campus Essen, and Research Alliance Ruhr, Research Center One Health University of Duisburg‐Essen Essen Germany; ^24^ Department of Dermatology, Comprehensive Cancer Center Erlangen – EMN Deutsches Zentrum Immuntherapie, Uniklinikum Erlangen Erlangen Germany

## Abstract

**Background:**

Adjuvant treatment with anti‐PD1 antibodies has been shown to effectively reduce the risk of recurrence in patients with resected metastatic melanoma. Whether a full 12‐month duration of treatment is needed to achieve full clinical benefit is not known. This study investigated the survival outcome depending on the duration of adjuvant anti‐PD1 therapy.

**Methods:**

From the prospective multicentre real‐world skin cancer registry ADOREG data of 620 patients who finished adjuvant treatment with nivolumab or pembrolizumab for AJCCv8 stage III/IV resected melanoma was analyzed. Recurrence‐free survival (RFS) and overall survival (OS) were compared between patients with regular treatment duration (52 ± 4 weeks; *n* = 229) and no disease recurrence during therapy (A_1_) and patients with a premature end of treatment (<48 weeks; *n* = 214, B). Patients with disease recurrence during adjuvant treatment were included in cohort A_2_.

**Results:**

The median duration of follow‐up was 26.0 months [interquartile range (IQR) 18.0–34.0] in group A_1_ [median treatment duration 51.3 weeks (IQR 50.0–52.1) and 19.0 months (IQR 13.0–29.0)] in group B [median treatment duration 22.2 weeks (IQR 10.0–34.8)]. Reasons for early discontinuation were treatment‐related side effects in 45.3% (*n* = 97) and other reasons than toxicity in 54.7% (*n* = 117). The 2‐year rate of RFS was 72.4% (95% CI, 68.5–76.3) for patients in group B and 51.5% (95% CI, 48.8–54.2) in patients with regular and intended regular treatment duration (A_1_ plus A_2_). When analysing the patients who did not relapse during adjuvant treatment (A_1_), there was a significantly higher RFS rate of 84.1% (95% CI, 81.5–86.7). When only assessing patients with a recurrence after more than 12 months after initiation of therapy, there was a trend towards better RFS in patients with regular treatment duration.

**Conclusions:**

In patients with resected metastatic melanoma, shorter treatment duration with anti‐PD1 antibodies is not associated with a worse outcome.

## Graphical abstract

This study investigated whether a shorter duration of adjuvant anti‐PD1 therapy in resected melanoma is associated with a worse outcome. Patients with a shorter treatment duration and who did not stop therapy due to disease recurrence have no shorter recurrence‐free and overall survival than patients with a full 12‐month treatment.

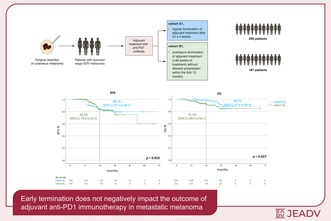

**Why was the study undertaken?**

- This study investigated whether a shorter duration of adjuvant anti‐PD1 therapy in resected melanoma is associated with a worse outcome.

**What does this study add?**

- Patients with a shorter treatment duration of adjuvant anti‐PD1 therapy and who did not stop therapy due to disease recurrence have no shorter recurrence‐free and overall survival than patients with a full 12‐month treatment.

**What are the implications of this study for clinical care?**

- Patients who develop adverse events during adjuvant anti‐PD1 therapy may not benefit from treatment continuation.

## INTRODUCTION

Patients with local, nodal or distant metastatic melanoma have a risk of recurrence and disease progression of 8%–29%, 47% and 86%, respectively, after complete resection.^1^ After excision of metastases with no evidence of disease in imaging scans, relapse is caused by residual cells, which are referred to as minimal residual disease.^2^ Relapse is not only associated with considerable stress^3^ but also with fatal disease courses. Melanoma‐specific survival at 5 years ranges from 80% to 93% for stage IIIA and from 30% to 32% for stage IIID patients with nodal involvement.^4^, ^5^

Recently, adjuvant treatment with anti‐PD1 antibodies in resected melanoma could show a decreased risk of recurrence by 35% for nivolumab compared to adjuvant treatment with ipilimumab in stage III and IV disease^6^ and by 43% for pembrolizumab compared to placebo in stage III disease.^7^ In addition, patients with resected melanoma harbouring a BRAF V600 mutation are eligible for adjuvant treatment with the BRAF inhibitor dabrafenib in combination with the MEK inhibitor trametinib, which reduces the risk of recurrence by 53% in stage III disease.^8^

In the metastatic setting, durable responses were observed even after the discontinuation of immune checkpoint inhibitor (ICI) therapy and, more specifically, in anti‐PD1 therapy.^9^, ^10^ In fact, in patients with a complete response of their metastatic melanoma due to therapy with pembrolizumab or nivolumab, less than 20% relapsed after a median follow‐up of 20 months; in partial responders and patients with stable disease, rates were 32% and 50%, respectively.^11^ Furthermore, patients who discontinued due to side effects during the induction phase did not show worse progression‐free survival (PFS) or overall survival (OS) than patients who continued ICI therapy.^12^ When the first clinical trials for ICI in cutaneous melanoma were designed, metastatic melanoma had hitherto been regarded as an incurable disease leading to death in almost all cases. Hence, the continuation of the investigational drugs was intended until disease progression, toxicity or a maximum of 2 years.^13^

Since oncological therapies pose a considerable risk for toxicity, including long‐term side effects,^14^, ^15^ time toxicity^16^ and a financial burden for the health system or patients depending on the country's legislation, optimal treatment duration is highly relevant.

Currently, two prospective trials are ongoing on treatment duration. In the DANTE trial, PFS of anti‐PD1 treatment for 12 months compared to at least 24 months^17^ is being investigated, while the rate of ongoing responses 24 months after discontinuation of PD1 blockade is going to be analysed in patients with confirmed complete response or partial response in the Safe Stop trial.^18^ Since these trials are event‐driven, interim and final analyses are not expected before 2027.

In recent and current adjuvant trials, the treatment period was set to 12 months, regardless of whether the investigated agents were nivolumab,^6^ pembrolizumab,^7^ combined relatlimab and nivolumab (RELATIVITY‐098, NCT05002569) or combined dabrafenib and trametinib.^8^ Discontinuation due to toxicity occurred in 9.7% for nivolumab, 13.8% for pembrolizumab and 26% for combined dabrafenib and trametinib. However, it was not clear from these trials whether patients who received shorter adjuvant treatment had a different outcome than patients who were treated for the full period of 12 months. Only 18.9% of patients treated with dabrafenib and trametinib relapsed after 12 months of adjuvant treatment compared to 39.1% of patients treated with pembrolizumab and 31.3% of patients treated with nivolumab.^6^, ^7^, ^8^ Given the different mechanism of action via blockade of the MAPK signalling pathway, the optimal duration of adjuvant treatment may be different for BRAF/MEK inhibition versus immune checkpoint inhibition.

The rationale for the choice of treatment duration in resected melanoma remains obscure, and it is unknown whether patients who stop their adjuvant therapy due to toxicity have the same outcome as patients who were treated for 12 months. Consequently, this study examines the relationship between the duration of treatment with adjuvant anti‐PD1 antibodies and disease outcome in resected metastatic cutaneous melanoma.

## METHODS

### Patient population

A total of 1238 patients with cutaneous melanoma stage III and IV AJCCv8 who underwent complete resection and received adjuvant treatment with the anti‐PD1 inhibitors nivolumab or pembrolizumab between 09/2015 and 01/2022 were identified from the prospective real‐world multicentre skin cancer registry ADOREG of the German Dermatologic Cooperative Oncology Group. Of those, only patients who had terminated adjuvant treatment were included in the analysis. Patients who received prior treatment with any other ICI, targeted therapy (BRAF‐inhibitor and/or MEK‐inhibitor) and/or blinded study medication were excluded. Patients with a follow‐up of less than 3 months were also excluded. The ADOREG was approved by the Medical Ethics Committee of the University Duisburg‐Essen (14‐5921‐BO), and written informed consent for participation was obtained from all patients.

**FIGURE 1:**
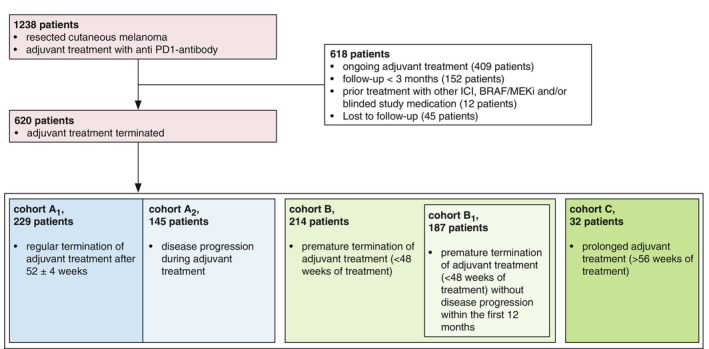
Study flow for patients with resected melanoma and adjuvant treatment. ICI, immune checkpoint inhibitors.

In total, 620 patients from 49 clinical centres were included and stratified into different cohorts. Cohort A_1_ (*n* = 229) included patients who completed 52 ± 4 weeks of adjuvant anti‐PD1 treatment. Patients with disease recurrence while on adjuvant treatment (*n* = 145) were considered intended standard treatment duration and therefore included in cohort A_2_ for additional analysis. Patients in cohort B (*n* = 214) terminated adjuvant anti‐PD1 therapy prematurely (treatment period of less than 48 weeks) for reasons other than disease progression, whereas patients in cohort C (*n* = 32) were kept on adjuvant treatment for longer than 56 weeks (Figure 1). To evaluate the efficacy of the different treatment durations (regular treatment duration versus premature termination), we compared (i) all patients with intended 1‐year adjuvant therapy including patients who relapsed during that year (cohort A_1_ + A_2_) with all patients who terminated earlier due to toxicity or the patient's preference (cohort B) and (ii) all patients without disease relapse within the first year with 12 months of adjuvant therapy (cohort A_1_) versus patients who terminated earlier (cohort B_1_). To evaluate the treatment efficacy of patients who received adjuvant therapy longer than 12 months, we compared patients with regular treatment duration (cohort A_1_) with prolonged treatment duration (cohort C, Table 1).

**TABLE 1:** Advantages and disadvantages of the different comparison approaches.

Compared groups	Cohort description	Limitation
Cohort A_1_ + A_2_ vs. cohort B	All patients were considered for the comparison	All patients with disease progression during treatment were included, leading to a selection bias in cohort A (including all patients who performed worse)
Cohort A_1_ vs. cohort B	Comparison between patients who have terminated adjuvant treatment without patients with disease progression during treatment	All patients with early disease recurrence were not considered, leading to a selection bias in cohort A (including all patients who had a good outcome)
Cohort A_1_ vs. cohort B_1_ (excluding patients with disease progression within 12 months)	Comparison between patients who have terminated adjuvant treatment and excluding patients with disease progression within 12 months after treatment start	Comparison between the two cohorts with the least selection bias but exclusion of many patients

### Statistical analysis

The adjuvant treatment initiation was defined as the index date. The follow‐up period was defined as the time from the end of adjuvant treatment until death, the last contact date or the end of the observation period (01/2022), whichever occurred first. The minimal follow‐up period after the end of adjuvant treatment was set to 3 months.

RFS was defined as the time of first recurrence of melanoma or death regardless of cause, whichever occurred first. OS was calculated from the index date of adjuvant treatment until death, the last contact date, or the end of the observation period (01/2022), whichever occurred first. Patients without recurrence or death before the data cut‐off were censored at the end of the observation period or at the last contact date, whichever applied first.

Continuous data are presented as median and interquartile ranges, and categorical data are presented as percentages. Median RFS and OS were estimated by Kaplan–Meier analysis and compared with log‐rank tests. Median follow‐up was defined as the median time between the start date of adjuvant immunotherapy and death or the last known alive date. *p* values < 0.05 were considered clinically significant. Confidence intervals for 2‐year RFS and OS rates were obtained.

Multivariate Cox proportional hazards regression analyses were performed to assess the impact of baseline patient and tumour characteristics on RFS. The following parameters were included in the multivariate analysis: sex, age, duration of adjuvant treatment, Breslow depths and ulceration status of the primary melanoma, BRAF and NRAS mutation status, and disease stage by AJCC v.8 (IIIA, IIIB, IIIC, IIID, IV). Statistical analyses were conducted with the statistical software SPSS Version 29.

## RESULTS

### Patients' characteristics

A total of 620 melanoma patients were included in the analysis. The median age in cohorts B and C was higher compared to cohort A_1_ (63.0 years vs. 60.0 years; 64.0 years vs. 60.0 years). In group A_2_, disease stages were higher (higher percentage of stage IIIC and IV patients) compared to the other groups, in which the distribution of the stages was comparable. In addition, rates of NRAS mutant melanoma were lower in cohort C compared to cohort A_1_ (6.3% vs. 18.3%). On the contrary, cohort B had a higher share of BRAF‐mutated melanoma patients compared to cohort A_1_ (36.4% vs. 26.2%). Tumour stages between the main comparison groups, A_1_ and B_1_, were similarly distributed. All cohorts were well balanced for sex and treatment (Table 2).

**TABLE 2:** Baseline characteristics of the patients in the three cohorts.

	Cohort A (A_1_ + A_2_)	Cohort A_1_	Cohort A_2_	Cohort B	Cohort B_1_	Cohort C
	Standard treatment duration + disease progression during therapy	Standard treatment duration without disease progression during therapy (52 ± 4 weeks)	Intended standard treatment duration with disease progression during therapy	Shorter treatment duration (<48 weeks)	Shorter treatment duration (<48 weeks) without disease progression within the first 12 months	Longer treatment duration (>56 weeks)
	(*n* = 374)	(*n* = 229)	(*n* = 145)	(*n* = 214)	(*n* = 187)	(*n* = 32)
Age, years						
Median	61.0	60.0	64.0	63.0	64.0	64.0
Interquartile range	51.0–70.0	51.0–69.0	53.0–73.0	53.0–73.0	53.0–73.0	68.5–77.5
Sex, no. (%)						
Male	216 (57.8)	135 (59.0)	81 (55.9)	111 (51.9)	94 (50.3)	20 (62.5)
Female	158 (42.2)	94 (41.0)	64 (44.1)	103 (48.1)	93 (49.7)	12 (37.5)
Resected stage, no. (%)						
IIIA	37 (9.9)	30 (13.1)	7 (4.8)	25 (11.7)	22 (11.7)	5 (15.6)
IIIB	132 (35.3)	91 (39.7)	41 (38.3)	72 (33.6)	64 (34.2)	7 (21.9)
IIIC	156 (41.7)	86 (37.6)	70 (48.3)	91 (42.5)	80 (42.8)	10 (31.2)
IIID	3 (0.8)	2 (0.9)	1 (0.7)	2 (1.0)	2 (1.0)	0 (0)
IV	46 (12.3)	20 (8.7)	26 (17.9)	24 (11.2)	19 (10.2)	10 (31.2)
Duration of adjuvant treatment, weeks						
Median		51.3	16.9	22.2	23.9	59.9
Interquartile range		50.0–52.1	10.0–25.9	10.0–34.8	10.6–35.5	56.8–89.5
Follow‐up, months						
Median		26.0	18.0	19.0	19.0	27.5
Interquartile range		18.0–34.0	12.0–31.0	13.0–29.0	13.0–29.0	23.0–38.0
Reason for discontinuation of adjuvant treatment, no. (%)						
Regular end of treatment	229 (61.2)	229 (100.0)				
Side effects/toxicity				97 (45.3)	88 (47.1)	
Other reasons (except toxicity)				117 (54.7)	99 (52.9)	
Patient's wish				82 (38.3)	73 (39.0)	
Progression of second cancer				3 (1.4)	2 (1.1)	
New severe disease (not related to ICI)				5 (2.3)	3 (1.6)	
Not specified				27 (12.6)	21 (11.2)	
Reason for longer treatment duration, no. (%)						
High risk of disease recurrence						14 (43.8)
Unintended treatment continuation						10 (31.3)
Not specified						8 (25.0)
Disease recurrence	145 (38.7)		145 (100.0)			
Not applicable						32 (100.0)
BRAF status, no. (%)						
Mutation	112 (29.8)	60 (26.2)	52 (35.9)	78 (36.4)	68 (36.4)	8 (25.0)
No mutation	189 (50.5)	120 (52.4)	69 (47.6)	95 (44.4)	81 (43.3)	13 (40.6)
Unknown	73 (19.5)	49 (21.4)	24 (16.6)	41 (19.2)	38 (20.3)	11 (34.4)
NRAS status, no. (%)						
Mutation	74 (19.8)	42 (18.3)	32 (22.1)	38 (17.8)	30 (16.0)	2 (6.3)
No mutation	109 (29.1)	65 (28.4)	44 (30.3)	64 (29.9)	55 (29.4)	6 (18.8)
Unknown	191 (51.1)	122 (53.3)	69 (47.6)	112 (52.3)	102 (54.5)	24 (75.0)
Treatment, no. (%)						
Pembrolizumab	126 (33.7)	73 (31.9)	53 (36.5)	79 (36.9)	69 (36.9)	14 (43.7)
Nivolumab	248 (66.3)	156 (68.1)	92 (63.4)	135 (63.1)	118 (63.1)	18 (56.3)

*Note*: Percentages are given in parenthesis. Groups A_1_ (highlighted in light blue) and B_1_ (highlighted in light green) were used for the main part of the analysis.

Multivariate Cox regression analysis identified the duration of adjuvant treatment, Breslow depth of the primary melanoma, BRAF and NRAS mutations, but not sex, age, ulceration status of the primary melanoma or disease stage by AJCC v.8 as independent prognostic factors of RFS (Table 3).

**TABLE 3:** Multivariate Cox regression analyses for RFS.

Parameters included (reference)	RFS HR (95% CI), *p* value
Sex (male)	
Male vs. female	1.254 (0.837–1.878), 0.272
Age	1.008 (0.993–1.022), 0.302
Duration of adjuvant treatment	**0.961 (0.950–0.072), <0.001**
Breslow depth of the primary melanoma	**1.036 (1.005–1.069), 0.022**
Ulceration status of the primary melanoma (no ulceration)	
No ulceration vs. ulceration	0.851 (0.527–1.374), 0.509
BRAF mutation status (wild‐type)	
Wild‐type vs. BRAF mutation	**2.268 (1.320–3.896), 0.003**
NRAS mutation status (wild‐type)	
Wild‐type vs. NRAS mutation	**1.877 (1.112–3.169), 0.018**
Disease stage by AJCCv8 (IIIA)	
IIIA vs. IIIB	1.063 (0.447–2.527), 0.891
IIIA vs. IIIC	1.917 (0.800–4.591), 0.144
IIIA vs. IIID	3.230 (0.360–28.983), 0.295
IIIA vs. IV	2.170 (0.832–5.664), 0.113

*Note*: Significant values are in bold.

### Regular versus shortened treatment duration including disease progression during treatment (cohorts A_1_ + A_2_ vs. cohort B)

**FIGURE 2:**
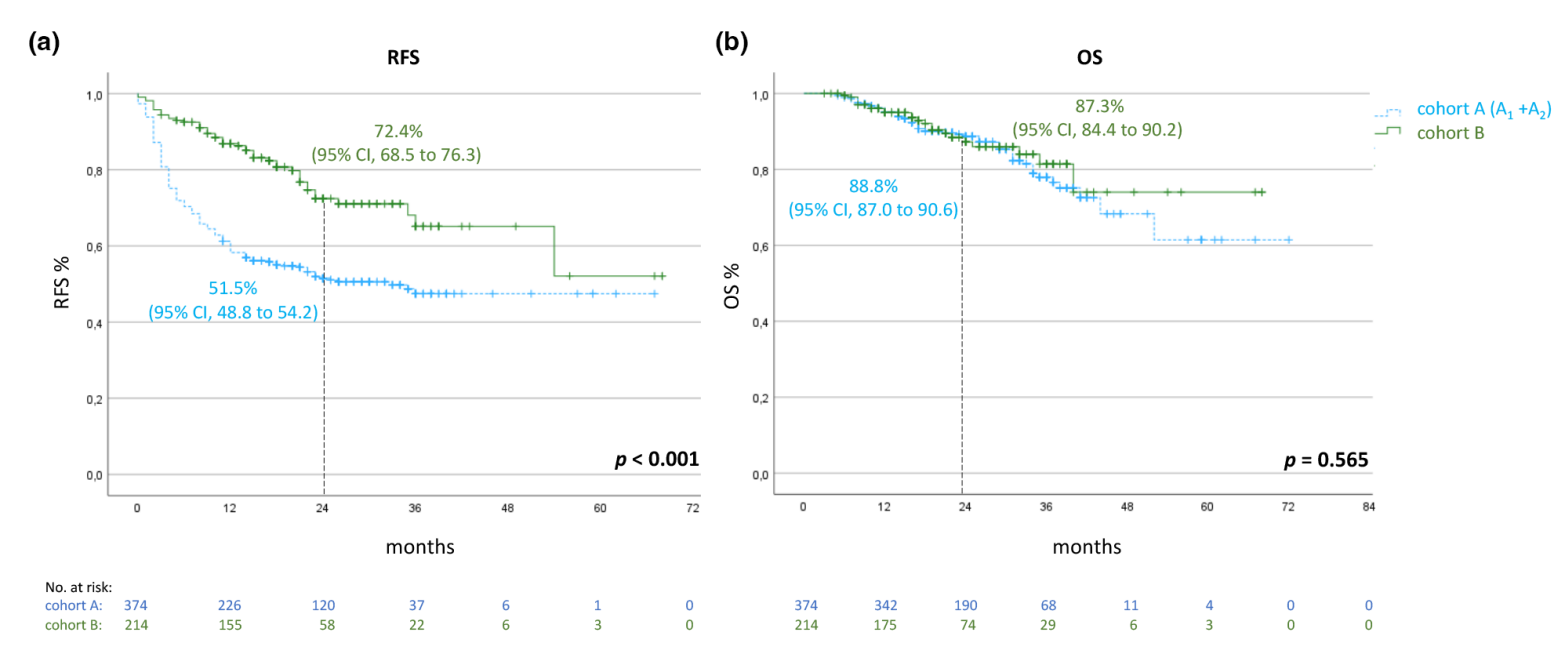
Kaplan–Meier estimates for RFS (a) and OS (b) of patients with a regular adjuvant treatment duration, including patients who relapsed during treatment (cohort A_1_ + A_2_; *n* = 374) and patients with a premature end of treatment (cohort B; *n* = 214). OS, overall survival; RFS, recurrence‐free survival.

At first, we compared all patients who were treated with adjuvant anti‐PD1 antibodies for 52 weeks (±4 weeks) including those patients with intended standard treatment duration, and who progressed during adjuvant therapy with those patients who had a shorter treatment duration of less than 48 weeks. Among all patients who were included in this analysis, 229 patients experienced a disease recurrence, and 80 patients have died. The 2‐year RFS rates were 72.4% (95% CI, 68.5–76.3) for patients who stopped adjuvant therapy prematurely compared to 51.5% (95% CI, 48.8 to 54.2) for patients who finished adjuvant treatment regularly after 52 weeks or who had progressed during treatment. The 2‐year OS rates were 87.3% (95% CI, 84.4–90.2) compared to 88.8% (95% CI, 87.0–90.6) (Figure 2).

When excluding patients with resected stage IIIA melanoma and a low recurrence rate, and patients with stage IIID and IV melanoma and a high recurrence rate, patients in group A (standard duration, *n* = 288) still had a 2‐year RFS of 51.3% (95% CI, 48.2–54.4) compared to 71.2% (95% CI, 66.4–76.0) in group B (shorter treatment duration, *n* = 163) and a 2‐year OS of 88.2% (95% CI, 86.2–90.2) compared to 85.9% (95 CI, 82.3–89.5), respectively.

### Regular versus shorter treatment duration excluding disease progression during therapy (cohort A_1_ vs. cohort B)

**FIGURE 3:**
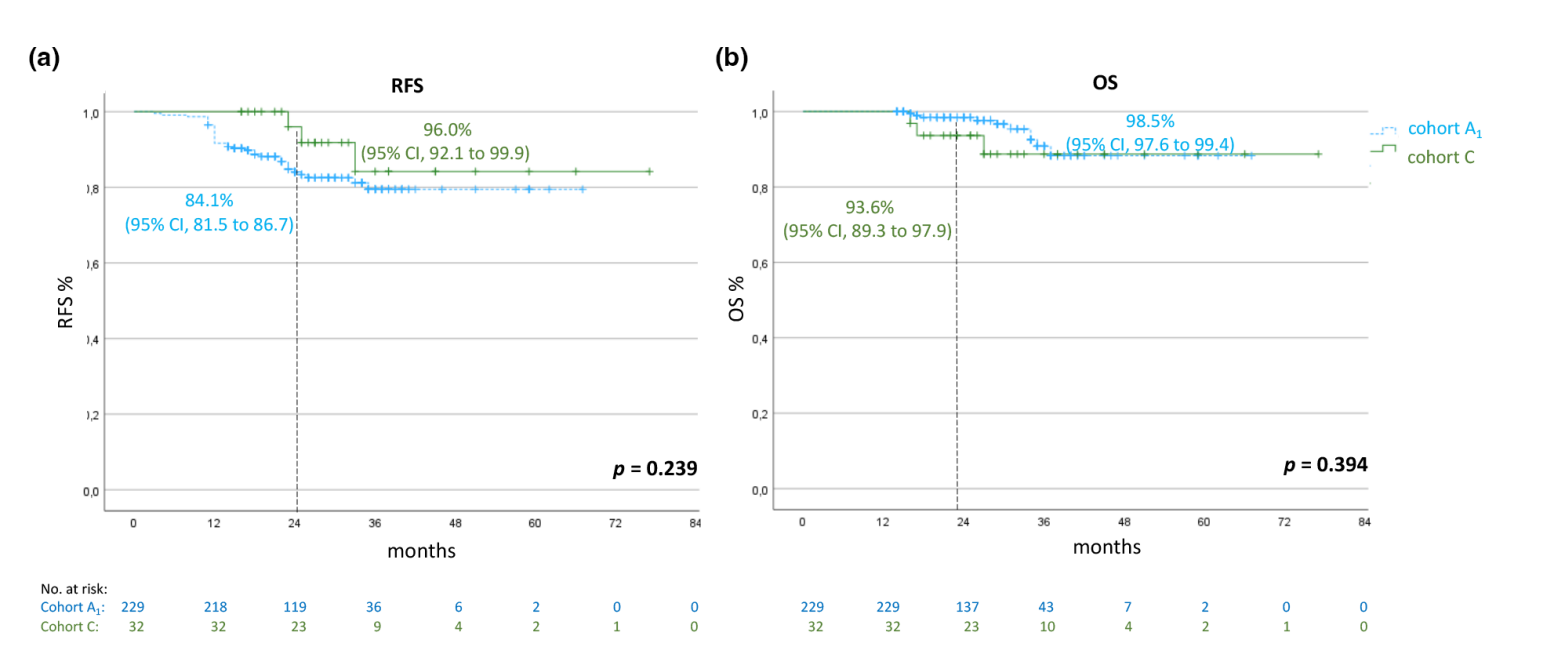
Kaplan–Meier estimates of RFS (a) and OS (b) of patients with regular treatment duration excluding patients who relapsed during treatment (cohort A_1_; *n* = 229) or who stopped treatment prematurely (cohort B; *n* = 214). OS, overall survival; RFS, recurrence‐free survival.

For further analysis, patients with intended standard treatment duration and relapse during adjuvant treatment were not considered. At the time of data closure, 36 patients who had stopped treatment regularly after 52 weeks (cohort A_1_) and 48 patients who had stopped treatment prematurely (cohort B) had a disease recurrence, and 33 patients had died. At 2 years, the rates for RFS were 84.1% (95% CI, 81.5–86.7) for cohort A_1_ and 72.4% (95% CI, 68.5 to 76.3) for cohort B. The rates for OS at 2 years were 98.5% (95% CI, 97.6–99.4) and 87.3% (95% CI, 84.4–90.2), respectively (Figure 3).

**FIGURE 4:**
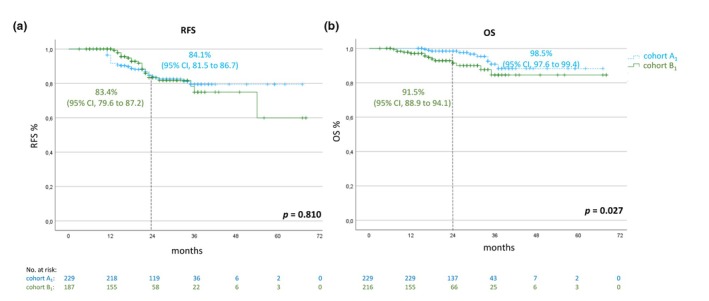
Kaplan–Meier estimates for RFS (a) and OS (b) of patients with a regular adjuvant treatment duration (cohort A_1_; *n* = 229) and patients with a premature end of treatment (cohort B_1_; *n* = 187). Patients who relapsed within the first year after initiation of adjuvant treatment were excluded from both cohorts. OS, overall survival; RFS, recurrence‐free survival.

Additionally, we compared patients from cohorts A_1_ and B_1_ (thus excluding all patients who had a relapse within the first 12 months after initiation of adjuvant therapy). Altogether, 57 relapses after a period of 12 months of adjuvant treatment and 24 deaths were documented. The 2‐year RFS rates were 84.1% (95% CI, 81.5 to 86.7) for cohort A_1_ and 83.4% (95% CI, 79.6–87.2) for cohort B_1_, respectively. The corresponding OS rates were 98.5% (95% CI, 97.6–99.4) compared to 91.5% (95% CI, 88.9–94.1; Figure 4) for cohorts A_1_ and B1, respectively.

### Regular versus prolonged treatment duration (cohort A_1_ vs. cohort C)

**FIGURE 5:**
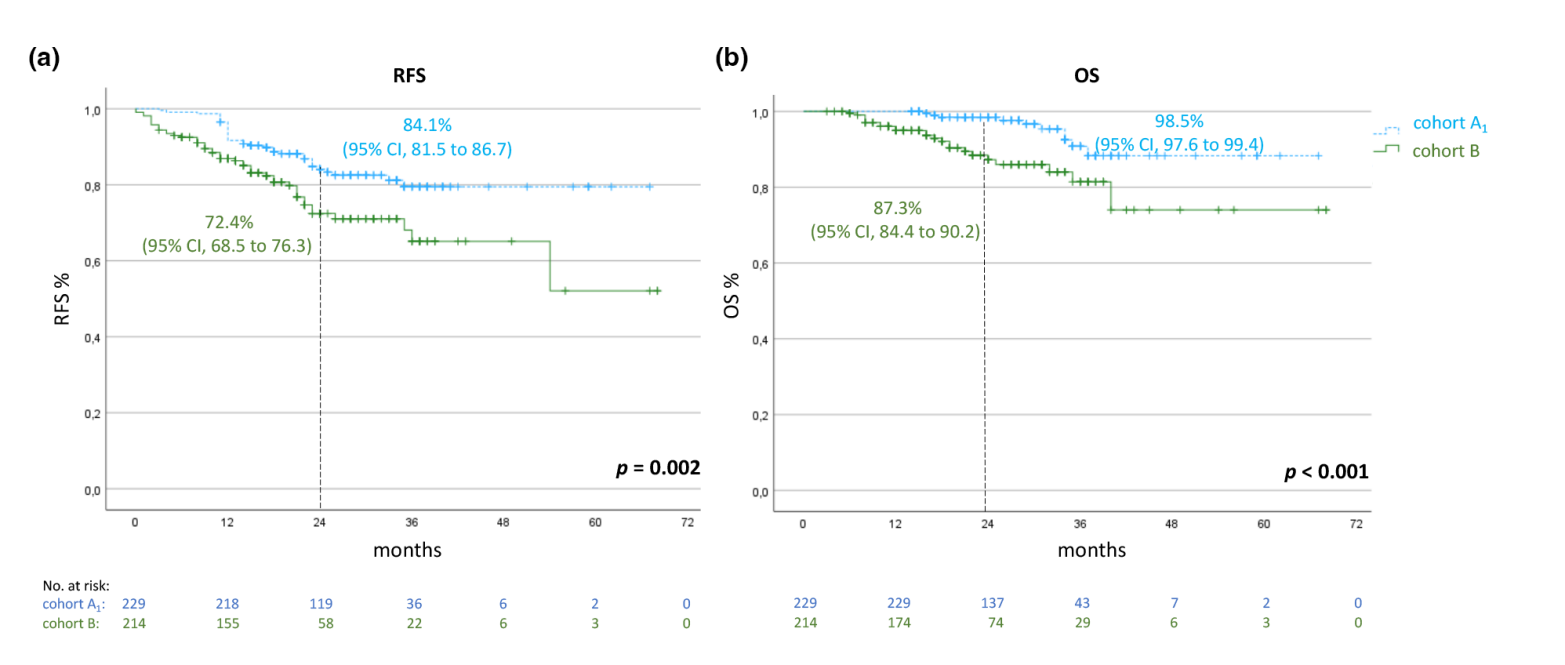
Kaplan–Meier estimates for RFS (a) and OS (b) of patients with a regular adjuvant treatment duration (cohort A1; *n* = 229) and patients with prolonged treatment (cohort C; *n* = 32). OS, overall survival; RFS, recurrence‐free survival.

In patients who received adjuvant treatment with an anti‐PD1 antibody for 52 ± 4 weeks (cohort A_1_) or longer (cohort C), a total of 39 disease relapses and 13 deaths occurred. The 2‐year RFS rates were 84.1% (95% CI, 81.5–86.7) for cohort A_1_ compared to 96.0% (95% CI, 92.1–99.9) for cohort C. The OS rates were 98.5% (95% CI, 97.6–99.4) for cohort A_1_ compared to 93.6% (95% CI, 89.3–97.9) for cohort C (Figure 5).

### Resected local disease versus resected distant metastatic disease

In patients who were treated after resection of local metastatic disease (stage III, *n* = 399), the 2‐year RFS rate was 78.7% (95% CI, 76.3–81.1) and the 2‐year OS rate was 94.4% (95% CI, 93.0–95.8) compared to 80.2% (95% CI, 73.8–86.6) and 87.5% (95% CI, 82.2–92.8), respectively, in patients who were treated after resection of distant metastatic disease (stage IV, *n* = 44).

We compared patients with resected local disease with respect to treatment duration. Patients who stopped treatment prematurely (*n* = 190) had a lower rate of RFS and OS after 2 years of 71.5% (95% CI, 67.2–75.8) and 88.0% (95% CI, 84.9–91.1), respectively, compared to 83.8% (95% CI, 81.0–86.6) and 98.8% (95% CI, 98.0–99.6)in patients who were treated for 52 ± 4 weeks (*n* = 209).

**FIGURE 6:**
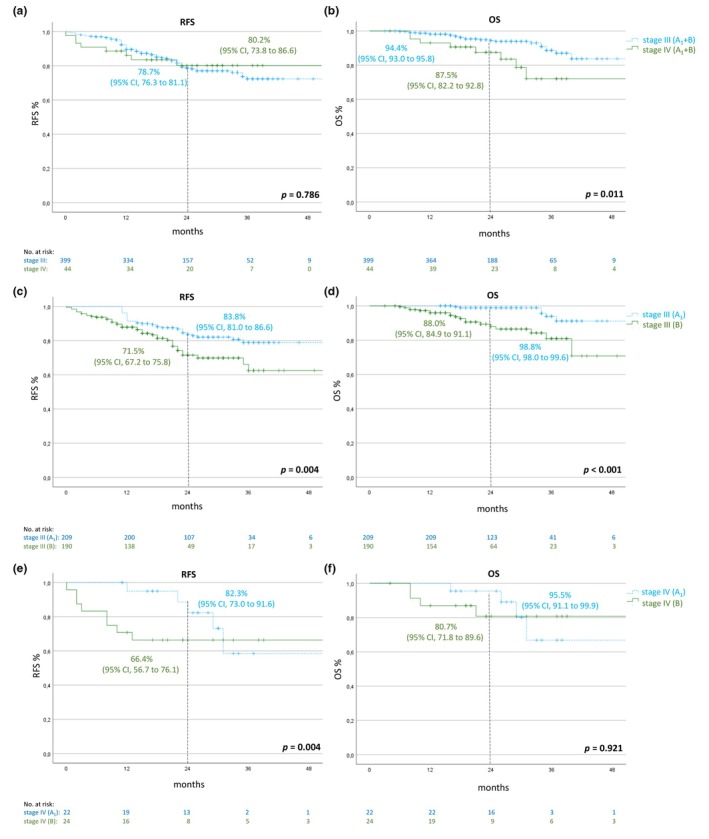
RFS and OS depending on stage and treatment duration. Panels a and b show RFS and OS of patients with any adjuvant treatment duration according to stage of disease (stage III vs. stage IV AJCC 2017). Panels c and d show RFS and OS of stage III patients with regular (cohort A_1_) vs. shorter (cohort B) duration of adjuvant therapy. Panels e and f show RFS and OS of stage IV patients with regular (cohort A_1_) vs. shorter (cohort B) duration of adjuvant therapy. Data are represented as Kaplan–Meier estimates. OS, overall survival; RFS, recurrence‐free survival.

In patients with resected distant metastatic melanoma, the 2‐year RFS and OS rates were also lower, with 66.4% (95% CI, 56.7–76.1) and 80.7% (95% CI, 71.8–89.6), respectively, in patients who ended adjuvant treatment early (*n* = 24) than in patients who received regular treatment (*n* = 22), with corresponding rates of 82.3% (95% CI, 73.0–91.6) and 95.5% (95% CI, 91.1–99.9; Figure 6), respectively.

### Early termination of adjuvant treatment due to toxicity versus other reasons than toxicity

**FIGURE 7:**
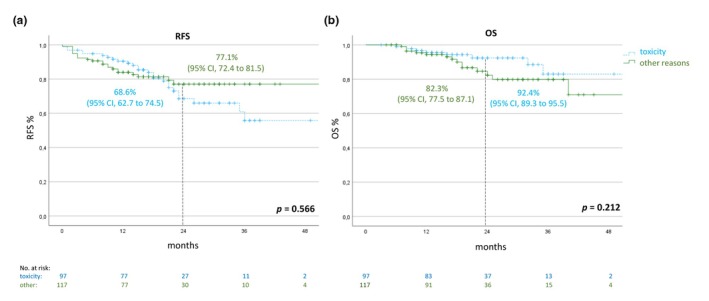
RFS and OS of patients with early termination of adjuvant therapy with anti‐PD1 (cohort B). Patients who stopped for toxicity were compared to patients who stopped for other reasons. OS, overall survival; RFS, recurrence‐free survival.

To investigate whether the reason for the premature end of adjuvant treatment has an influence on outcome, we compared RFS and OS rates between patients who stopped therapy due to toxicity (*n* = 97) with patients who terminated early for other reasons than toxicity (*n* = 117). RFS rates were 68.6% (95% CI, 62.7–74.5) and 77.1% (95% CI, 72.4–81.8) of patients with early termination due to toxicity compared to discontinuation for other reasons, respectively. The 2‐year OS rate was higher in patients who stopped due to toxicity at 92.4% (95% CI, 89.3–95.5) compared to 82.3% (95% CI, 77.5–87.1) of patients who stopped early for other reasons (Figure 7).

To determine whether the time point of toxicity that had led to discontinuation of adjuvant treatment had an influence on RFS and OS, we compared patients with early toxicity (onset earlier or equal than 3 months after beginning of adjuvant treatment; *n* = 36) with patients with late toxicity (onset later than 3 months after beginning of adjuvant treatment; *n* = 61). The 2‐year RFS rates were similar at 65.4% (95% CI, 56.1–74.7) and 70.3% (95% CI, 62.6–78.0). The 2‐year OS rates were 86.5% (95% CI, 80.1–92.9) and 96.3% (95% CI, 93.7–98.9), respectively.

## DISCUSSION

This study shows that in patients who terminated adjuvant treatment with pembrolizumab or nivolumab earlier than 48 weeks for reasons other than disease progression, RFS or OS were not significantly inferior compared to patients who either ended their adjuvant treatment regularly after a period of 52 ± 4 weeks or who terminated treatment due to disease progression. To diminish the confounding influence of the patients who recurred while on treatment, patients with a low risk of recurrence (resected stage IIIA) and patients with a high risk of recurrence (resected stages IIID and IV) were excluded for parts of the analysis without any significant impact on RFS or OS. Comparison of patients who relapsed after 12 months showed a trend for improved RFS in patients who were treated for the whole intended period compared to patients with a shorter treatment duration. However, there was no difference in OS. In patients who stayed on adjuvant anti‐PD1 antibody therapy for longer than 52 ± 4 weeks, there was a trend of a higher OS rate, but the patient cohort was too small (*n* = 32) to show a statistical significance. Patients with resected stage III melanoma showed a more pronounced benefit from longer adjuvant treatment compared to patients with resected stage IV disease, who had only a tendency to improve RFS and OS with longer treatment duration.

Data on optimal treatment duration in adjuvant therapy are lacking. However, in metastatic melanoma patients, durable complete responses after a single dose of ICI therapy have been reported.^19^ In a neoadjuvant trial with a single dose of anti‐PD1 therapy, a complete or major pathologic response, which was defined as the detection of viable tumour cells of less than 10%, was achieved in 30% of patients and all eight patients had no recurrence after continuation of adjuvant treatment.^20^ In patients with a major pathologic response in their largest lymph node after two doses of neoadjuvant ipilimumab 1 mg/kg and nivolumab 3 mg/kg, therapeutic lymph node dissection and adjuvant therapy were even omitted in the PRADO trial,^21^ nonetheless, this led to a high RFS of 93% (*n* = 60). Given the fact that modes of action of MAPK inhibition differ from those of ICI, the optimal treatment duration of adjuvant BRAF/MEK inhibitor therapy might also be different from that of ICI therapy. A large retrospective multicenter study, which investigated 1198 melanoma patients who received adjuvant treatment, included 195 patients with BRAF/MEK inhibitor therapy.^22^ With a median treatment duration of 8.0 months, the RFS of 86.5% at 12 months for patients with dabrafenib and trametinib therapy was significantly higher compared to 74.1% for patients who were treated with either pembrolizumab or nivolumab, with a similar median treatment duration of 7.0 and 9.0 months, respectively. In addition, in this study, ulceration status and a higher disease stage were also associated with disease recurrence. However, in our analysis, risk factors for RFS included Breslow depth of the primary tumour, BRAF and NRAS mutation, whereas ulceration status and higher disease stage were not significantly associated with recurrence.

According to our data, patients with a shorter adjuvant treatment duration due to toxicity have no worse outcome than patients who were treated for the intended period of 12 months. A comparison of the reasons for premature discontinuation of adjuvant treatment showed that patients who discontinued treatment due to ICI‐induced toxicities tended to have a higher OS rate than patients who discontinued treatment for other reasons. In our analysis, there was no difference between patients who experienced toxicities either within or later than 3 months after initiation of adjuvant treatment. This is in accordance with data showing that patients who develop immune‐related adverse events in the non‐adjuvant setting have a better treatment efficacy.^23^, ^24^ According to our data, when toxicity leads to early termination, patients do not have a worse outcome than patients who discontinue adjuvant treatment for other reasons, and they may not benefit from continuation of adjuvant therapy.

Limitations of the study are that the data come from a prospective multicentre registry, and treatment duration was not assigned. The data quality from the ADOREG registry might be impaired as only central monitoring and no local monitoring had been performed. Reasons for longer adjuvant treatment duration and premature discontinuation of adjuvant treatment were not specified in all cases. Another limitation is a small sample size of patients who received longer adjuvant treatment and who had resected stage IV melanoma. To overcome these limitations, further prospective translational studies are necessary to better understand optimal treatment duration in the adjuvant setting.

## CONCLUSIONS

In patients with resected metastatic melanoma stages III and IV, shorter treatment duration with anti‐PD1 antibodies is not associated with a worse outcome.

## AUTHOR CONTRIBUTIONS

DT: Conceptualization, data curation, investigation, validation, visualization, writing – original draft. EL: data curation, project administration, supervision, writing – review and editing. CL: data curation, investigation, writing – review and editing. MK: data curation, investigation, writing – review and editing. UL: data curation, project administration, supervision, writing – review and editing. BS: data curation, investigation, writing – review and editing. PT: data curation, investigation, writing – review and editing. JH: data curation, investigation, writing – review and editing. MS: data curation, investigation, writing – review and editing. JU: data curation, investigation, writing – review and editing. ED: data curation, investigation, writing – review and editing. FM: data curation, investigation, writing – review and editing. CP: data curation, investigation, writing – review and editing. AK: data curation, investigation, writing – review and editing. RH: data curation, investigation, writing – review and editing. MW: data curation, project administration, supervision, writing – review and editing. LZ: data curation, investigation, writing – review and editing. FM: data curation, investigation, writing – review and editing. RR: data curation, investigation, writing – review and editing. PM: data curation, investigation, writing – review and editing. FB: data curation, investigation, writing – review and editing. IW: data curation, investigation, writing – review and editing. RG: data curation, project administration, supervision, writing – review and editing. DS: data curation, investigation, writing – review and editing. CB: data curation, project administration, supervision, writing – review and editing. SU: data curation, project administration, supervision, writing – review and editing. LH: data curation, project administration, supervision, writing – review and editing.

## FUNDING INFORMATION

None.

## CONFLICT OF INTEREST STATEMENT

DT reports consultancy, speaker fees, research or travel grants: BMS, Roche, Sanofi, Recordati, Kyowa Kirin, Sun Pharma, Pierre Fabre. EL reports consultancy, speaker fees, research or travel grants: MSD, Novartis, BMS, Pierre Fabre, Sun Pharma, Takeda, Sanofi. CL reports consultancy, speaker fees, research or travel grants: BMS, MSD, Merck, Roche, Immunocore, Novartis, Pierre Fabre, Sanofi, Sun Pharma, Almirall, Kyowa Kirin, Biontech. UL reports consultancy, speaker fees, research or travel grants: Sun Pharma, Regeneron, Sanofi, Almirall, Novartis, MSD, Pierre Fabre. BS reports consultancy, speaker fees, research or travel grants: Novartis, Immunocore, Sanofi, Pierre Fabre. PT reports consultancy, speaker fees, research or travel grants: Almirall, Biotest, BMS, Sanofi, L'Oréal, Pierre Fabre, Merck Serono, Kyowa Kirin, Biofrontera, 4SC. FM reports consultancy, speaker fees, research or travel grants: Pierre Fabre, Novartis, Sun Pharma, BMS. CP reports consultancy, speaker fees, research or travel grants: BMS, MSD, Novartis, Sun Pharma, Amgen, Sanofi, Abbvie, Kyowa Kirin. LZ reports consultancy, speaker fees, research or travel grants: MSD, BMS, Novartis, Pierre Fabre, Sun Pharma, Sanofi. FM reports consultancy, speaker fees, research or travel grants: Novartis, Roche, BMS, MSD, Pierre Fabre, Sanofi. PM reports consultancy, speaker fees, research or travel grants: MSD, Novartis, BMS, Pierre Fabre, Sanofi, Roche, Beiersdorf, Amgen, Almirall, Sun Pharma, Regeneron, Delcath, Immunocore. RG reports consultancy, speaker fees, research or travel grants: Amgen, Sanofi, Merck, Kyowa‐Kirin, Almirall, Sun Pharma, Recordati, BMS, Novartis, MSD, Pierre‐Fabre, Delcath, Immunocore, Boehringer‐Ingelheim, 4SC. SU reports consultancy, speaker fees, research or travel grants: BMS, MSD, Merck Serono, Novartis, IGEA Clinical Biophysics, Pierre Fabre, Sun Pharma. LH reports consultancy, speaker fees, research or travel grants: BMS, MSD, Merck, Roche, Amgen, Curevac, Novartis, Sanofi, Pierre Fabre. The other authors have declared no conflict of interest.

## ETHICAL APPROVAL

The ADOREG was approved by the Medical Ethics Committee of the University Duisburg‐Essen (14‐5921‐BO).

## ETHICS STATEMENT

The patients in this manuscript have given written informed consent for participation in the ADOREG and for publication of their case details.

## Data Availability

The data that support the findings of this study are available from the corresponding author upon reasonable request.

## References

[1] Leeneman B , Franken MG , Coupé VMH , Hendriks MP , Kruit W , Plaisier PW , et al. Stage‐specific disease recurrence and survival in localized and regionally advanced cutaneous melanoma. Eur J Surg Oncol. 2019;45(5):825–831. 30765270 10.1016/j.ejso.2019.01.225

[2] Rambow F , Rogiers A , Marin‐Bejar O , Aibar S , Femel J , Dewaele M , et al. Toward minimal residual disease‐directed therapy in melanoma. Cell. 2018;174(4):843–855.e19. 10.1016/j.cell.2018.06.025 30017245

[3] Herschbach P , Dinkel A . Fear of progression. Recent Results Cancer Res. 2014;197:11–29. 24305766 10.1007/978-3-642-40187-9_2

[4] Gershenwald JE , Scolyer RA , Hess KR , Sondak VK , Long GV , Ross MI , et al. Melanoma staging: evidence‐based changes in the American joint committee on cancer eighth edition cancer staging manual. CA Cancer J Clin. 2017;67(6):472–492. 29028110 10.3322/caac.21409 PMC5978683

[5] Garbe C , Keim U , Suciu S , Amaral T , Eigentler TK , Gesierich A , et al. Prognosis of patients with stage III melanoma according to American joint committee on cancer version 8: a reassessment on the basis of 3 independent stage III melanoma cohorts. J Clin Oncol. 2020;38(22):2543–2551. 32530760 10.1200/JCO.19.03034 PMC7392743

[6] Larkin J , Del Vecchio M , Mandalá M , Gogas H , Arance Fernandez AM , Dalle S , et al. Adjuvant nivolumab versus ipilimumab in resected stage III/IV melanoma: 5‐year efficacy and biomarker results from CheckMate 238. Clin Cancer Res. 2023;29(17):3352–3361. 37058595 10.1158/1078-0432.CCR-22-3145 PMC10472092

[7] Eggermont AMM , Blank CU , Mandala M , Long GV , Atkinson VG , Dalle S , et al. Longer follow‐up confirms recurrence‐free survival benefit of adjuvant pembrolizumab in high‐risk stage III melanoma: updated results from the EORTC 1325‐MG/KEYNOTE‐054 trial. J Clin Oncol. 2020;38(33):3925–3936. 32946353 10.1200/JCO.20.02110 PMC7676886

[8] Dummer R , Hauschild A , Santinami M , Atkinson V , Mandalà M , Kirkwood JM , et al. Five‐year analysis of adjuvant dabrafenib plus trametinib in stage III melanoma. N Engl J Med. 2020;383(12):1139–1148. 32877599 10.1056/NEJMoa2005493

[9] Topalian SL , Sznol M , McDermott DF , Kluger HM , Carvajal RD , Sharfman WH , et al. Survival, durable tumor remission, and long‐term safety in patients with advanced melanoma receiving nivolumab. J Clin Oncol. 2014;32(10):1020–1030. 24590637 10.1200/JCO.2013.53.0105 PMC4811023

[10] Robert C , Ribas A , Hamid O , Daud A , Wolchok JD , Joshua AM , et al. Durable complete response after discontinuation of pembrolizumab in patients with metastatic melanoma. J Clin Oncol. 2018;36(17):1668–1674. 29283791 10.1200/JCO.2017.75.6270

[11] Jansen YJL , Rozeman EA , Mason R , Goldinger SM , Geukes Foppen MH , Hoejberg L , et al. Discontinuation of anti‐PD‐1 antibody therapy in the absence of disease progression or treatment limiting toxicity: clinical outcomes in advanced melanoma. Ann Oncol. 2019;30(7):1154–1161. 30923820 10.1093/annonc/mdz110

[12] Schadendorf D , Wolchok JD , Hodi FS , Chiarion‐Sileni V , Gonzalez R , Rutkowski P , et al. Efficacy and safety outcomes in patients with advanced melanoma who discontinued treatment with nivolumab and ipilimumab because of adverse events: a pooled analysis of randomized phase II and III trials. J Clin Oncol. 2017;35(34):3807–3814. 28841387 10.1200/JCO.2017.73.2289 PMC5791828

[13] Marron TU , Ryan AE , Reddy SM , Kaczanowska S , Younis RH , Thakkar D , et al. Considerations for treatment duration in responders to immune checkpoint inhibitors. J Immunother Cancer. 2021;9(3):e001901. 33653801 10.1136/jitc-2020-001901 PMC7929825

[14] Schulz TU , Zierold S , Sachse M , Pesch G , Tomsitz D , Schilbach K , et al. Long‐term consequences of checkpoint inhibitor therapy: prevalence and impact on patients' health related quality of life. Eur J Cancer. 2022;176:88–99. 36198246 10.1016/j.ejca.2022.08.029

[15] Patrinely JR Jr , Young AC , Quach H , Williams GR , Ye F , Fan R , et al. Survivorship in immune therapy: assessing toxicities, body composition and health‐related quality of life among long‐term survivors treated with antibodies to programmed death‐1 receptor and its ligand. Eur J Cancer. 2020;135:211–220. 32599411 10.1016/j.ejca.2020.05.005 PMC7374019

[16] Gupta A , Eisenhauer EA , Booth CM . The time toxicity of cancer treatment. J Clin Oncol. 2022;40(15):1611–1615. 35235366 10.1200/JCO.21.02810

[17] Coen O , Corrie P , Marshall H , Plummer R , Ottensmeier C , Hook J , et al. The DANTE trial protocol: a randomised phase III trial to evaluate the duration of ANti‐PD‐1 monoclonal antibody treatment in patients with metastatic melanoma. BMC Cancer. 2021;21(1):761. 34210290 10.1186/s12885-021-08509-w PMC8246129

[18] Mulder EEAP , de Joode K , Litière S , Ten Tije AJ , Suijkerbuijk KPM , Boers‐Sonderen MJ , et al. Early discontinuation of PD‐1 blockade upon achieving a complete or partial response in patients with advanced melanoma: the multicentre prospective safe stop trial. BMC Cancer. 2021;21(1):323. 33765967 10.1186/s12885-021-08018-w PMC7993897

[19] Matull J , Livingstone E , Wetter A , Zimmer L , Zaremba A , Lahner H , et al. Durable complete response in a melanoma patient with unknown primary, associated with sequential and severe multi‐organ toxicity after a single dose of CTLA‐4 plus PD‐1 blockade: a case report. Front Oncol. 2020;10:592609. 33262949 10.3389/fonc.2020.592609 PMC7686558

[20] Huang AC , Orlowski RJ , Xu X , Mick R , George SM , Yan PK , et al. A single dose of neoadjuvant PD‐1 blockade predicts clinical outcomes in resectable melanoma. Nat Med. 2019;25(3):454–461. 30804515 10.1038/s41591-019-0357-y PMC6699626

[21] Reijers ILM , Menzies AM , van Akkooi ACJ , Versluis JM , van den Heuvel NMJ , Saw RPM , et al. Personalized response‐directed surgery and adjuvant therapy after neoadjuvant ipilimumab and nivolumab in high‐risk stage III melanoma: the PRADO trial. Nat Med. 2022;28(6):1178–1188. 35661157 10.1038/s41591-022-01851-x

[22] Schumann K , Mauch C , Klespe KC , Loquai C , Nikfarjam U , Schlaak M , et al. Real‐world outcomes using PD‐1 antibodies and BRAF + MEK inhibitors for adjuvant melanoma treatment from 39 skin cancer centers in Germany, Austria and Switzerland. J Eur Acad Dermatol Venereol. 2023;37(5):894–906. 36433688 10.1111/jdv.18779

[23] Dimitriou F , Staeger R , Ak M , Maissen M , Kudura K , Barysch MJ , et al. Frequency, treatment and outcome of immune‐related toxicities in patients with immune‐checkpoint inhibitors for advanced melanoma: results from an institutional database analysis. Cancers (Basel). 2021;13(12):2931. 34208218 10.3390/cancers13122931 PMC8230729

[24] Hussaini S , Chehade R , Boldt RG , Raphael J , Blanchette P , Maleki Vareki S , et al. Association between immune‐related side effects and efficacy and benefit of immune checkpoint inhibitors – a systematic review and meta‐analysis. Cancer Treat Rev. 2021;92:102134. 33302134 10.1016/j.ctrv.2020.102134

